# SWI/SNF complex gene variations are associated with a higher tumor mutational burden and a better response to immune checkpoint inhibitor treatment: a pan-cancer analysis of next-generation sequencing data corresponding to 4591 cases

**DOI:** 10.1186/s12935-022-02757-x

**Published:** 2022-11-12

**Authors:** Yue Li, Xinhua Yang, Weijie Zhu, Yuxia Xu, Jiangjun Ma, Caiyun He, Fang Wang

**Affiliations:** Department of Molecular Diagnostics, State Key Laboratory of Oncology in South China, Collaborative Innovation Center for Cancer Medicine, Sun Yat-Sen University Cancer Center, 651 Dongfeng East Road, Yuexiu District, Guangzhou, 510060 China

**Keywords:** SWI/SNF complex genes, Mutational landscape, Tumor mutational burden, Microsatellite instability, Immune checkpoint inhibitors, Synthetic lethality

## Abstract

**Background:**

Genes related to the SWItch/sucrose nonfermentable (SWI/SNF) chromatin remodeling complex are frequently mutated across cancers. SWI/SNF-mutant tumors are vulnerable to synthetic lethal inhibitors. However, the landscape of SWI/SNF mutations and their associations with tumor mutational burden (TMB), microsatellite instability (MSI) status, and response to immune checkpoint inhibitors (ICIs) have not been elucidated in large real-world Chinese patient cohorts.

**Methods:**

The mutational rates and variation types of six SWI/SNF complex genes (*ARID1A*, *ARID1B*, *ARID2*, *SMARCA4*, *SMARCB1*, and *PBRM1*) were analyzed retrospectively by integrating next-generation sequencing data of 4591 cases covering 18 cancer types. Thereafter, characteristics of SWI/SNF mutations were depicted and the TMB and MSI status and therapeutic effects of ICIs in the SWI/SNF-mutant and SWI/SNF-non-mutant groups were compared.

**Results:**

SWI/SNF mutations were observed in 21.8% of tumors. Endometrial (54.1%), gallbladder and biliary tract (43.4%), and gastric (33.9%) cancers exhibited remarkably higher SWI/SNF mutational rates than other malignancies. Further, *ARID1A* was the most frequently mutated SWI/SNF gene, and *ARID1A* D1850fs was identified as relatively crucial. The TMB value, TMB-high (TMB-H), and MSI-high (MSI-H) proportions corresponding to SWI/SNF-mutant cancers were significantly higher than those corresponding to SWI/SNF-non-mutant cancers (25.8 vs. 5.6 mutations/Mb, 44.3% vs. 10.3%, and 16.0% vs. 0.9%, respectively; all *p * < 0.0001). Furthermore, these indices were even higher for tumors with co-mutations of SWI/SNF genes and *MLL2/3*. Regarding immunotherapeutic effects, patients with SWI/SNF variations showed significantly longer progression-free survival (PFS) rates than their SWI/SNF-non-mutant counterparts (hazard ratio [HR], 0.56 [95% confidence interval {CI} 0.44–0.72]; *p *< 0.0001), and *PBRM1* mutations were associated with relatively better ICI treatment outcomes than the other SWI/SNF gene mutations (HR, 0.21 [95% CI 0.12–0.37]; *p* = 0.0007). Additionally, patients in the SWI/SNF-mutant + TMB-H (HR, 0.48 [95% CI 0.37–0.54]; *p * < 0.0001) cohorts had longer PFS rates than those in the SWI/SNF-non-mutant + TMB-low cohort.

**Conclusions:**

SWI/SNF complex genes are frequently mutated and are closely associated with TMB-H status, MSI-H status, and superior ICI treatment response in several cancers, such as colorectal cancer, gastric cancer, and non-small cell lung cancer. These findings emphasize the necessity and importance of molecular-level detection and interpretation of SWI/SNF complex mutations.

**Supplementary Information:**

The online version contains supplementary material available at 10.1186/s12935-022-02757-x.

## Background

Precision diagnostics are prerequisites for achieving the goal of cancer precision treatment. The concept that cancer is a genetically driven disease is widely supported by therapeutic successes directed at particular mutations or pathways. The traditional paradigm of drug development in oncology has gradually shifted to a tissue-agnostic therapeutic model, wherein patients are deemed eligible for a given treatment based on the presence of specific molecular variations rather than on the cancer type (i.e., the affected tissue) [1]. One particularly representative class of tissue-agnostic drugs is tropomyosin receptor kinase (TRK) inhibitors, which have been approved for cancer treatment owing to their durable responses in diverse adult and pediatric cancer patients with *NTRK* fusions; moreover, various other potential tissue-agnostic drugs are being developed [2]. Notably, genes of the SWItch/sucrose nonfermentable (SWI/SNF) chromatin remodeling complex are potential candidates for tissue-agnostic drug development, as these genes are commonly mutated in 20–25% of all human cancers [3]; this prevalence is notably higher than that of *NTRK* fusions (0.3%) [4].

The SWI/SNF complex is an ATP-consuming multi-subunit cellular machine that modulates chromatin compaction, thereby, regulating DNA-related processes, such as transcription, replication, and repair [5]. There are three subfamilies of the SWI/SNF complex in mammals, namely, the BRG1/BRM-associated factor (BAF), polybromo-associated BAF (PBAF), and noncanonical BAF (ncBAF) complexes [6]. Among dozens of SWI/SNF complex genes, *ARID1A*, *ARID1B*, *ARID2*, *SMARCA4*, and *PBRM1* have been reported to be altered in  ≥ 5% of a certain tumor and are mutated above the background mutation rate in two or more cancer types, accounting for their gene length, which suggests that mutations in these genes are “driver” rather than “passenger” variations [7]. In addition, the biallelically inactivated *SMARCB1* was frequently found in malignant rhabdoid tumors, which is a clear evidence that at least one SWI/SNF subunit is indeed a tumor suppressor [8, 9]. According to our preliminary data, the median variant allele frequencies (VAFs) of these six genes were 13.3–17.2% (Additional file 1: Figure S1), consistent with the previous report. Therefore, we decided to focus on the above six genes in this study. AT-rich interactive domain 1A (*ARID1A*), also known as BAF250a, is a tumor suppressor that is typically mutated in Epstein-Barr virus-positive and microsatellite instability-high (MSI-H) gastric cancer [10, 11], ovarian clear cell carcinoma [12], endometrial cancer [13], and non-small cell lung cancer [14]. Further, *SMARCA4* (BRG1) encodes a core catalytic component of the SWI/SNF complex and its inactivation is indicative of the presence of hypercalcemic-type small cell carcinoma of the ovary [15, 16], and loss-of-function (LOF) mutations of *SMARCB1* (SNF5/INI1/BAF47), which encodes another core subunit of the SWI/SNF complex, have been identified in the majority of rhabdoid tumors [17, 18]. Furthermore, the loss of both *ARID1B* and *SMARCB1* expression has been detected in approximately one-third of undifferentiated endometrial cancers [19]. *ARID2* has also been identified as one of the most frequently altered genes in non-small cell lung cancer [20], gallbladder cancer [21], and metastatic breast cancer [22], and its deficiency can hamper DNA repair processes and enhance the sensitivity of lung cancer cells to DNA-damaging agents [23]. *PBRM1* encodes polybromo 1, a specific subunit of the PBAF complex, and reportedly, in clinical practice, *PBRM1* LOF variations favor the therapeutic effect of immune checkpoint inhibitors (ICIs) in renal cell carcinomas [24–26]. In addition to the aforementioned associations with various cancers, accumulating evidence suggests that SWI/SNF mutations can induce certain molecular perturbations in a synthetic lethal pattern [3, 27, 28], highlighting their potential as targets for drug development.

Next-generation sequencing (NGS) has been extensively applied as a cost-effective diagnostic tool in clinical practice and trials [29]. In the present study, we aimed to retrospectively integrate the NGS data corresponding to a large real-world Chinese patient cohort to comprehensively depict the landscape of SWI/SNF mutations and explored the associations between SWI/SNF variations and tumor mutational burden (TMB), MSI status, and therapeutic responses to ICIs across solid tumors. These findings can serve as a useful reference as well as a basis for molecular diagnostics and targeted drug development.

## Methods

### Study design and patient information

NGS data and clinical information corresponding to patients who visited the Department of Molecular Diagnostics of Sun Yat-sen University Cancer Center (Guangzhou, China) for NGS analysis between September 1, 2019 and June 30, 2021 were retrospectively included. All the cancer diagnoses were also confirmed via pathological examination. Cases with genomic alterations in at least one of the six SWI/SNF complex genes (*ARID1A*, *ARID1B*, *ARID2*, *SMARCA4*, *SMARCB1*, and *PBRM1*) were classified under the SWI/SNF-mutant group. Thereafter, NGS data, including variant genes, number of variants, variation types, protein changes, TMB value, TMB status, and MSI status, as well as clinical characteristics of the patients, including age, sex, smoking status, cancer type, TNM stage, ICI type, and progression-free survival (PFS) during ICI treatments, were systematically collected. The use of clinical and NGS data was approved by the Ethics Committee of the Sun Yat-Sen University Cancer Center (Approval number B2020-344-01). All the patients also provided written informed consent, and the study was performed in accordance with the Declaration of Helsinki.

### NGS and data processing

The detailed experimental steps and data analysis strategies for NGS were as previously described [30–32]. For library construction, approximately 0.5 μg of DNA fragments were mixed with Illumina-indexed adapters (Illumina, San Diego, CA, USA) using the KAPA Library Preparation Kit (Kapa Biosystems, Wilmington, MA, USA). A hybrid captured-based NGS assay covering approximately 1.1 megabases (Mb) of the genomic sequences of 1021 cancer-related genes (GenePlus-Beijing, China) was used for the sequencing, which was performed using a GenePlus 2000 sequencing system (Beijing, China) with 2 × 100 bp paired-end reads. DNA samples from matched peripheral white blood cells were sequenced simultaneously to filter out benign single nucleotide polymorphisms and possible germline mutations.

The sequencing data were then analyzed by aligning the clean reads to the reference human genome (hg38) using BWA18 (version 0.7.12-r1039) [33], and small insertions and deletions (indels) and single-nucleotide variants were identified using MuTect19 (version 1.1.4) [34]. A somatic mutation was confirmed if it was consistently detected in five high-quality reads (Phred score  ≥ 30, mapping quality  ≥ 30, and no paired-end read bias) and had a variant allele frequency  ≥ 1% [35]. Copy number variations (CNVs) were detected using the Copy Number Targeted Resequencing Analysis (CONTRA) software (http://contra-cnv.sourceforge.net/) [36], and mutations were then annotated to the genes using the ANNOVAR20 software (http://www.openbioinformatics.org/annovar/) [37].

### Classification of LOF and non-LOF variations

LOF variations generally include frameshift indels, nonsense mutations, and splice site mutations. Missense mutations can result in both LOF and non-LOF consequences. To properly stratify LOF/non-LOF mutations, we assessed all of the missense mutations using prediction scores MetaLR and MetaSVM for mutation pathogenicity analysis [38]. MetaLR and MetaSVM are ensemble models based on 10 component scores (SIFT, PolyPhen-2 HDIV, PolyPhen-2 HVAR, GERP  +  + , MutationTaster, Mutation Assessor, FATHMM, LRT, SiPhy, and PhyloP) and the maximum frequency observed in the 1000 genomes populations. Furthermore, these two ensemble scores have been reported to outperform all of their component scores [39]. Both scores range from 0 to 1, with scores close to 1 indicating certainty that the variant is deleterious. In this study, a missense mutation with both MetaLR and MetaSVM scores of  > 0.8 was classified as a LOF mutation as previously recommended [40].

### TMB and MSI evaluation

TMB was defined as the number of somatic nonsynonymous mutations/Mb of coding DNA (including small indels and single-nucleotide variants with a variant allele frequency  ≥ 3%). TMB values  ≥ 20 mutations/Mb in colorectal cancer were classified as TMB-high (TMB-H) [31], this cut-off was decided by the TMB values of 122 MSI-H colorectal cancer samples verified using a PCR assay by Geneplus. TMB values  ≥ 7.68 mutations/Mb in all the other cancers were classified as TMB-H according to the top quartile corresponding to 3234 samples (except for colorectal cancer) in the current study [41]. The MSI status was analyzed using MSIsensor (version 0.5). Specifically, MSI scores were calculated as the percentage of unstable somatic microsatellite loci in predefined microsatellite regions covered by the NGS panel used; a sample was determined to have MSI-H if the score was  > 8% (this cut-off was decided upon by Geneplus after comparing MSI results of NGS and PCR assay for five mononucleotide microsatellite loci, including NR-21, BAT-25, MONO-27, NR-24, and BAT-26).

### Statistical analysis

The response to immunotherapy was characterized by determining PFS, overall response rate (ORR), and disease control rate (DCR), which were explored based on data corresponding to the subset of patients that received ICI treatment. Specifically, PFS was calculated from the start date of the ICI treatments to the date of disease progression or last follow-up. The clinical characteristics of the SWI/SNF-mutant and SWI/SNF-non-mutant groups were compared using the chi-square test. Additionally, differences in TMB values between the two groups were assessed by performing the Mann–Whitney test, while co-occurring and mutually exclusive events were detected by performing the pair-wise Fisher exact test [42]. The possible biological functions and downstream signaling pathways related to all the mutated genes in 1001 SWI/SNF-mutant samples were explored using the Gene Ontology (GO) database [43, 44]. Survival curves and estimates of the median PFS were generated using the Kaplan–Meier methods and compared across different groups by performing the log-rank tests. Hazard ratios (HR) and 95% confidence intervals (CI) were also reported. Statistical significance was based on two-tailed tests at *p* < 0.05. GraphPad Prism (version 8.4.0, GraphPad Software, San Diego, CA, USA) was used for statistical analyses.

## Results

### Patient characteristics

A total of 4591 Chinese patients with 18 types of solid tumors were included in this study, and 21.8% of them carried variants in at least one of the six selected SWI/SNF genes (*ARID1A*, *ARID1B*, *ARID2*, *SMARCA4*, *SMARCB1*, and/or *PBRM1*). Among them, 301 patients with SWI/SNF variants (SWI/SNF-mutant group) and 700 patients without SWI/SNF variants (SWI/SNF-non-mutant group) had received ICIs, including anti-PD-1, PD-L1, and CTLA4 or their combinations. The SWI/SNF-non-mutant group had a higher proportion of patients with TNM stage I than the SWI/SNF-mutant group, while age, sex, smoking status, and ICI type were not markedly different between the two groups (Table 1).

**Table 1:** The clinical information of the study population grouped by whether carrying SWI/SNF variations

	Total			Treated by ICIs		
Characteristics	SWI/SNF-mutant	SWI/SNF-non-mutant	*p* value	SWI/SNF-mutant	SWI/SNF-non-mutant	*p* value
No. of patients	1001	3590		301	700	
Age at the diagnosis, median (range, years)	56 (14–90)	56 (1–89)		57 (14–87)	55 (9–85)	
≥ 55	572	1961	0.1611	157	361	0.8904
< 55	429	1629		144	339	
Sex						
Male	517	1748	0.1002	187	394	0.0938
Female	484	1842		114	306	
TNM stage						
I	140	629	< 0.0001	12	30	0.1377
II	202	502		64	109	
III	269	951		106	245	
IV	364	1396		119	316	
Unknow	26	112		0	0	
Smoking history						
Current/Former	178	668	0.5542	63	168	0.3263
Never	797	2810		238	532	
Unknow	26	112		0	0	
ICI types						
Anti PD-1				284	658	0.2511
Anti PD-L1				11	33	
Anti PD-1 + CTLA4				6	6	
Anti PD-1 + PD-L1				0	3	

*CTLA4* cytotoxic T lymphocyte-associated protein 4, *ICIs* immune checkpoint inhibitors, *PD-1* programmed death-1, *PD-L1* programmed death-ligand 1, *SWI/SNF* SWItch/sucrose nonfermentable

The most common cancer type observed in this study was non-small cell lung cancer (32.3%), followed by colorectal cancer (29.6%) and ovarian and fallopian tube cancer (7.9%). The top five malignancies with the highest SWI/SNF mutation rates were endometrial cancer (54.1%), gallbladder and biliary tract cancer (43.4%), gastric cancer (33.9%), urothelial cancer (30.6%), and ovarian and fallopian tube cancer (23.9%). The SWI/SNF mutation rate corresponding to the “Other” subset, which comprised some relatively uncommon tumors, including skin squamous cancer, urachal cancer, gastrointestinal stromal tumor, glioma, adrenal tumors, and medullary thyroid cancer, among others, was 18.6%.

### Spectrum of SWI/SNF complex genomic variations

Among the six SWI/SNF genes, *ARID1A* and *SMARCB1* were, respectively, the most and least frequently mutated genes in the majority of the cancer types (*ARID1A*, 10.7%; *SMARCA4*, 6.0%; *ARID1B*, 4.7%; *ARID2*, 4.0%; *PBRM1*, 3.5%; *SMARCB1*, 1.3%; Table 2, Additional file 1: Figure S1a). Notably, *SMARCA4* mutations were slightly more common than *ARID1A* mutations in cases of non-small cell lung cancer, cervical cancer, and melanoma. Interestingly, up to 25.0% of the SWI/SNF-mutant tumors showed genetic aberrations at two or more SWI/SNF genes (Table 2).

**Table 2:** Mutation rate of SWI/SNF complex genes in different cancer types

Cancer type	n	n/Total (%)	SWI/SNF mutation rate (%)	ARID1A (%)	ARID1B (%)	ARID2 (%)	PBRM1 (%)	SMARCA4 (%)	SMARCB1 (%)	≥ 2 Genes (n)	≥ 2 Genes (%)
Breast cancer	86	1.9	16.3	9.3	4.7	3.5	0	1.2	1.2	3	3.5
Cervical cancer	118	2.6	12.7	1.7	2.5	2.5	2.5	4.2	1.7	2	1.7
colorectal cancer	1358	29.6	23.0	12.1	6.8	5.0	5.4	6.8	1.8	115	8.5
Endometrial cancer	122	2.7	54.1	48.4	18.0	10.7	10.7	13.1	4.9	33	27.0
Esophagus cancer	37	0.8	21.6	8.1	5.4	5.4	0	0	2.7	2	5.4
Gallbladder and Biliary tract cancer	53	1.2	43.4	26.4	13.2	11.3	3.8	9.4	1.9	7	13.2
Gastric cancer	168	3.7	33.9	25.0	4.8	2.4	2.4	7.7	1.8	11	6.5
Kidney cancer	34	0.7	20.6	14.7	0	2.9	2.9	2.9	0	1	2.9
Liver cancer	84	1.8	17.9	8.3	2.4	2.4	7.1	3.6	1.2	3	3.6
Melanoma	119	2.6	12.6	2.5	3.4	3.4	3.4	3.4	0	3	2.5
Non-small cell lung cancer	1485	32.3	18.9	5.8	3.4	3.4	2.2	6.2	0.8	43	2.9
Other	242	5.3	18.6	7.0	1.2	4.1	4.5	6.2	1.7	11	4.5
Ovarian and Fallopian tube cancer	364	7.9	23.9	14.8	2.5	1.4	2.2	5.5	0.5	10	2.7
Pancreatic cancer	93	2.0	17.2	9.7	0	3.2	1.1	2.2	1.1	0	0.0
Prostatic cancer	19	0.4	15.8	10.5	5.3	5.3	0	5.3	5.3	1	5.3
Small cell lung cancer	20	0.4	15.0	0	0	10.0	0	5.0	0	0	0.0
Soft tissue sarcoma	127	2.8	11.0	3.9	1.6	3.9	0.8	1.6	0.8	2	1.6
Urothelial cancer	62	1.4	30.6	21.0	8.1	1.6	0	6.5	0	3	4.8
Total	4591	100.0	21.8	10.7	4.7	4.0	3.5	6.0	1.3	250	5.4

*N* number, *SWI/SNF* switch/sucrose nonfermentable

**Fig. 1:**
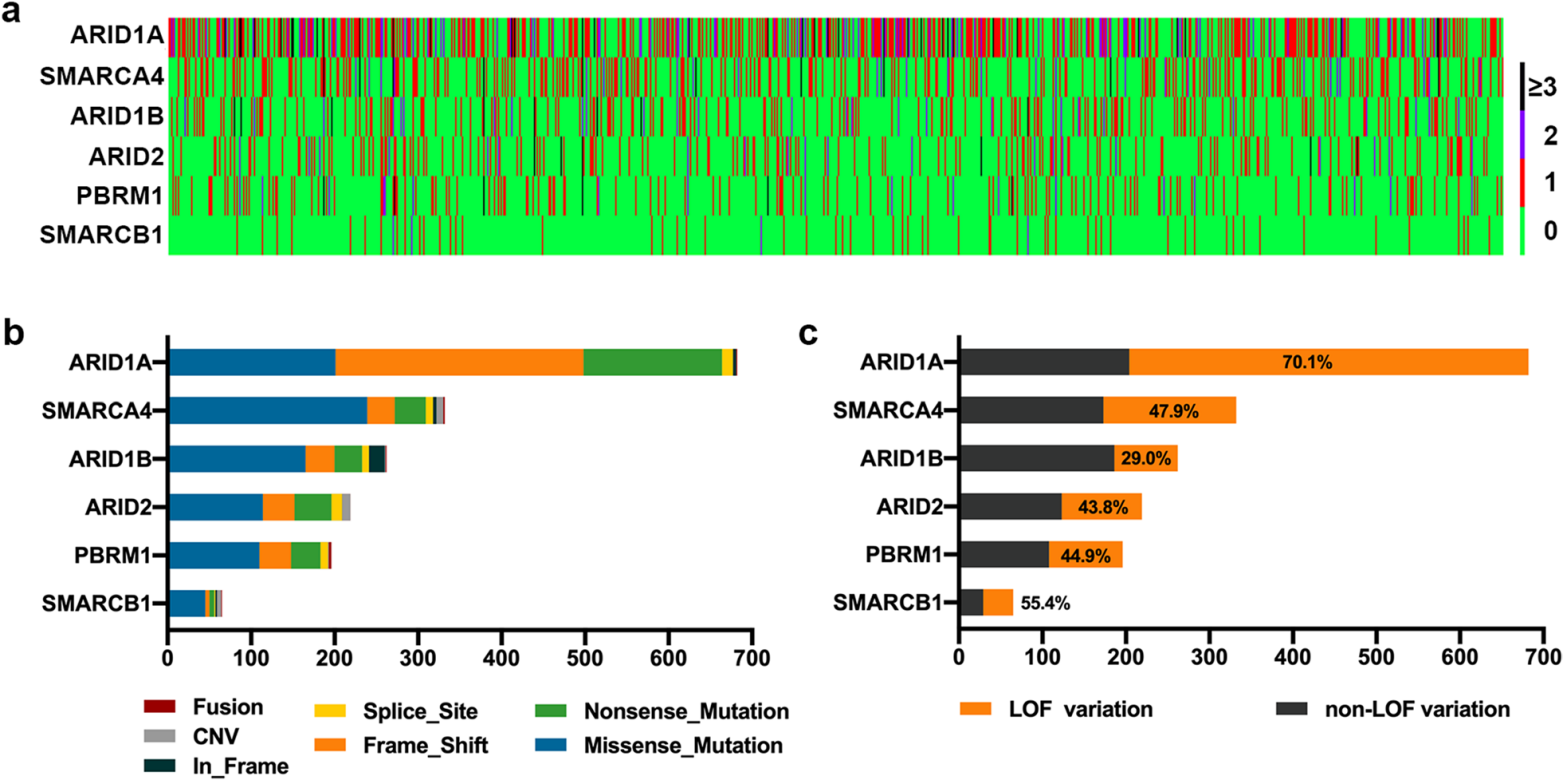
The mutational spectrum of six SWI/SNF genes. **a** The number and distribution of six SWI/SNF gene variations in 1001 tumor samples. **b** The composition of variation types in each SWI/SNF gene. **c** The ratio of loss-of-function (LOF) mutations in each SWI/SNF gene

All the genetic alterations were classified under the following seven types: frameshift indels, in-frame indels, nonsense mutations, missense mutations, splice site mutations, CNVs, and fusions (gene rearrangements). Frameshift indels constituted the most common variation type in *ARIDIA*, whereas missense mutations were more common in the other five genes (Additional file 1: Figure S1b). The proportions of LOF mutations of the SWI/SNF complex genes were as follows: *ARID1A*, 70.1%; *ARID2*, 43.8%; *PBRM1*, 44.9%; *ARID1B*, 29.0%; *SMARCA4*, 47.9%; and *SMARCB1*, 55.4% (Fig. 1c).

**Fig. 2:**
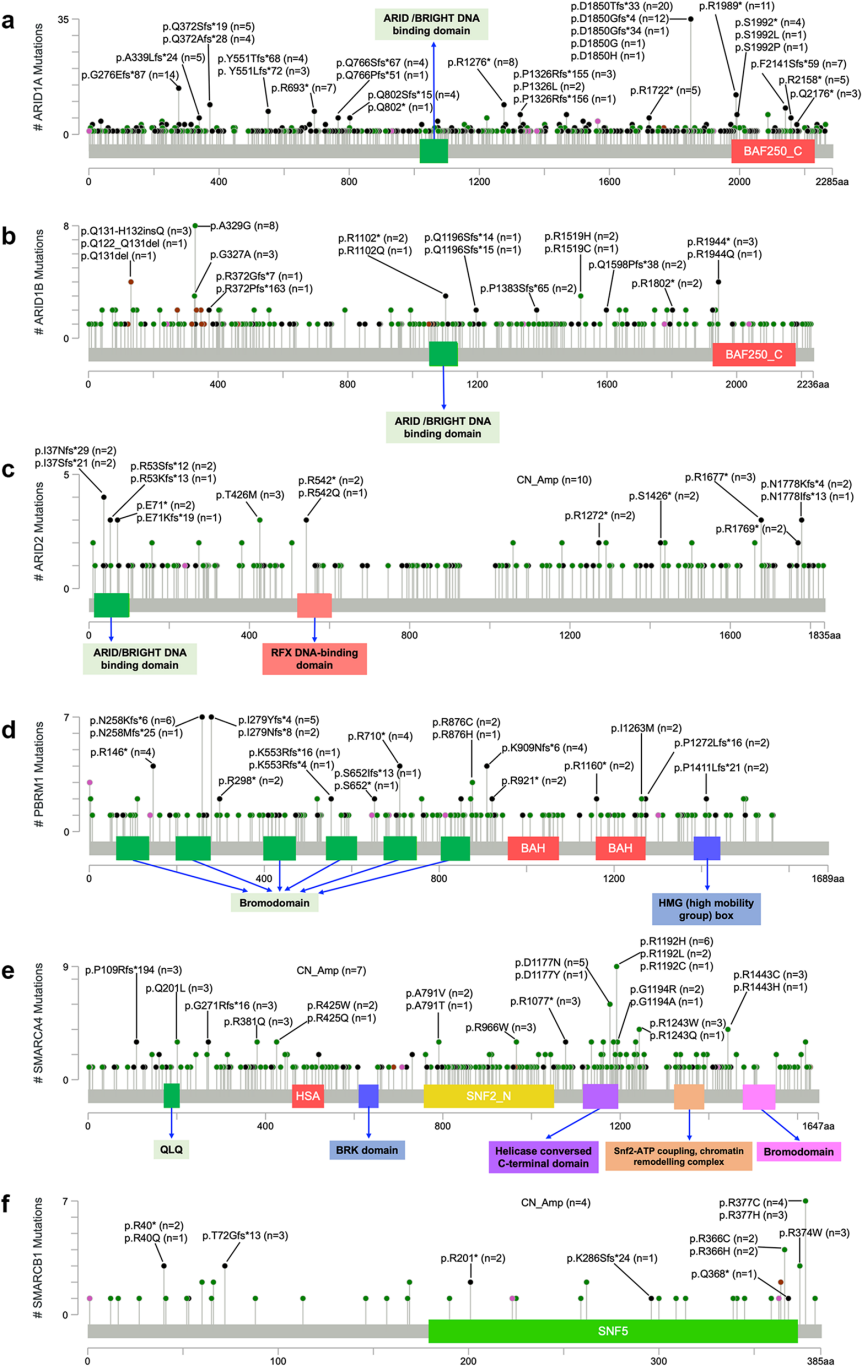
The distribution of frequently-detected mutations of SWI/SNF genes. The amino acid changes and frequencies of relatively frequently-detected mutations of each gene were annotated by using MutationMapper (http://www.cbioportal.org/mutation_mapper)

Although most variations were widely distributed along the full length of each gene, a number of frameshift indels (fs) and nonsense mutations (*), which led to the truncation of protein products, were relatively frequently detected. These included D1850fs, G276fs, R1989*, R1276*, and F2141fs in *ARID1A* (Fig. 2a); R1944* in *ARID1B* (Fig. 2b); I37fs, R53fs, and p.E71* in *ARID2* (Fig. 2c); N258fs, I279fs, p.R146, R710*, and K909fs in *PBRM1* (Fig. 2d); P109fs, G271fs, and R1077* in *SMARCA4* (Fig. 2e); and R40*, T72fs, and R201* in *SMARCB1* (Fig. 2f). In addition, several missense mutations, such as A329G in *ARID1B*, R1192H/L/C and D1177N/Y in *SMARCA4*, and R366C/H and R377C/H in *SMARCB1*, were detected in a relatively greater number of cases (Fig. 2b, e, and f).

### Co-occurrence and mutual exclusivity

**Fig. 3:**
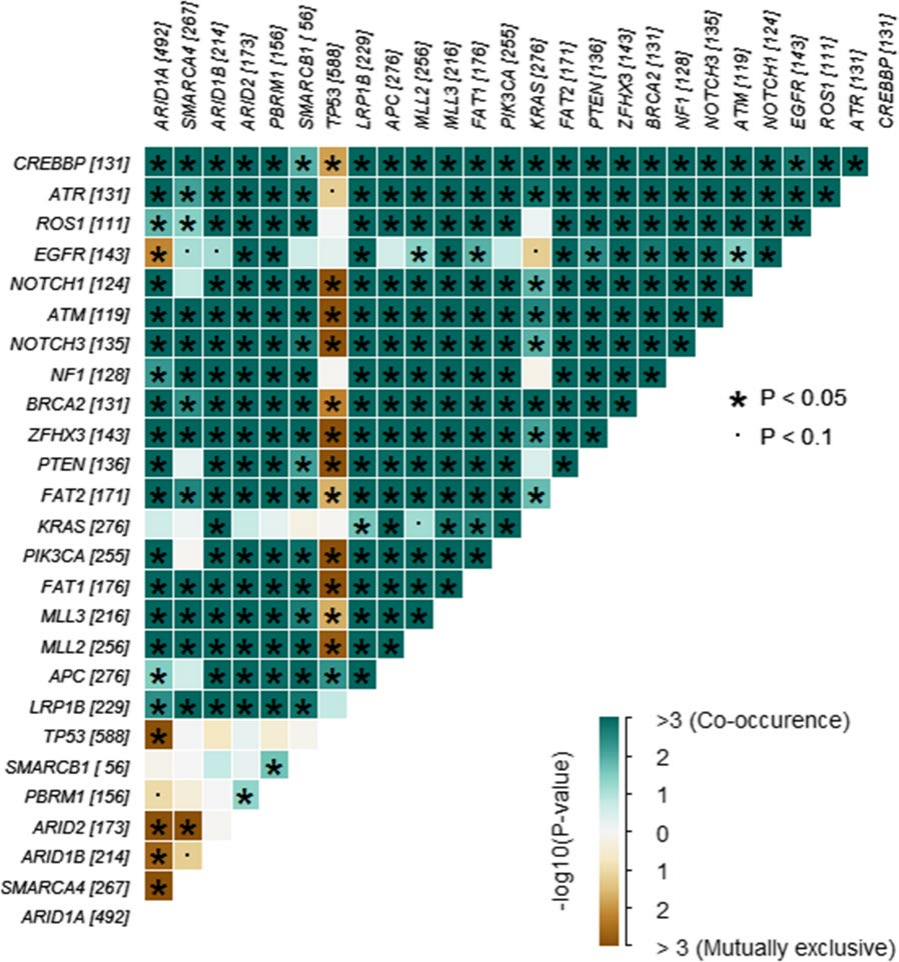
The co-occurrence and exclusivity of mutations of SWI/SNF genes and accompanied top 20 mutated genes. The value corresponding to each gene is the number of samples with mutation(s) of that gene. * *p* < 0.05, ·*p* < 0.1

To uncover the potential pattern of SWI/SNF gene mutations, the co-occurrence and exclusivity of the mutations of the six SWI/SNF genes and the top 20 most frequently altered genes across all tumors were explored. Of the top 20 mutated genes, excluding the six hub genes, it is well known that *APC*, *KRAS*, *PIK3CA*, *EGFR*, *LRP1B*, *BRCA2*, *ATM*, and *ROS1* are mutated in several cancer types, such as non-small cell lung cancer, colorectal cancer, and endometrial cancer. In this study, we observed that *ARID1A* was the second most frequently mutated gene following *TP53*. Furthermore, *ARID1A* variations were identified in a mutually exclusive pattern with variations in *EGFR*, *TP53*, *ARID1B*, *ARID2*, and *SMARCA4*; and *ARID2* variations were identified in a mutually exclusive pattern with variations in *SMARCA4*. However, *PBRM1* tended to co-mutate with *ARID2* and *SMARCB1* (Fig. 3). *ARID1A*, as well as the well-established tumor suppressor, *PTEN*, and the oncogene, *PIK3CA*, showed significant variations, mutually exclusive of *TP53*, suggesting that *ARID1A* may be a functional driver like *PTEN* and *PIK3CA*.

Notably, *MLL2* (*MLL4*/*KMT2D*) and *MLL3* (*KMT2C*), belonging to a family of mammalian histone H3 lysine 4 (H3K4) methyltransferases [45], were frequently co-mutated with SWI/SNF genes (Fig. 3). Reportedly, KMT2D collaborates with the SWI/SNF complex to promote cell type-specific enhancer activation [46], and cancer cells with KMT2C deficiency have higher endogenous DNA damage and genomic instability [47]. The subset carrying both SWI/SNF and *MLL2*/*3* mutations showed higher average TMB values (*MLL2*, 70.9 mutations/Mb; *MLL3*, 74.5 mutations/Mb), TMB-H ratios (*MLL2*, 80.5%; *MLL3*, 83.6%), and MSI-H ratios (*MLL2*, 48.6%; *MLL3*, 46.6%) than the whole SWI/SNF-mutant group (all *p* < 0.0001).

### Association of SWI/SNF mutations with TMB and MSI

Previous studies have revealed the existence of a potential linkage between the SWI/SNF chromatin remodeling complex and DNA repair, TMB, and MSI [6]. Thus, in this study, these relationships were further analyzed. Our results indicate that the average TMB value corresponding to SWI/SNF-mutant tumors was markedly higher than that corresponding to SWI/SNF-non-mutant tumors, regardless of the cancer type (25.8 vs. 5.6 mutations/Mb, *p * < 0.0001). The TMB-H and MSI-H ratios corresponding to SWI/SNF-mutant tumors were also significantly higher than those corresponding to the SWI/SNF-non-mutant tumors (TMB-H ratio: 44.3% vs. 10.3%, *p * < 0.0001; MSI-H ratio: 16.0% vs. 0.9%, *p * < 0.0001), even though the differences were not significant for certain malignancies, such as kidney cancer, pancreatic cancer, prostate cancer, and urothelial cancer. SWI/SNF-mutant colorectal cancer, endometrial cancer, and gastric cancer exhibited both higher TMB-H and MSI-H ratios than their SWI/SNF-non-mutant counterparts (Table 3). Furthermore, the patient group with mutations at two or more SWI/SNF genes had significantly higher TMB values (69.0 vs. 11.3 mutations/Mb, *p * < 0.0001), TMB-H ratios (86.2% vs. 40.5%, *p * < 0.0001), and MSI-H ratios (48.0% vs. 5.3%, *p * < 0.0001) than those with mutations in a single SWI/SNF gene.

**Table 3:** The associations of SWI/SNF mutations with TMB and MSI status in different malignancies

Cancer type	Average TMB value (mutations/Mb)			TMB-H proportion			MSI-H proportion		
	SWI/SNF-non-mutant	SWI/SNF-mutant	*p*	SWI/SNF-non-mutant	SWI/SNF-mutant	*p*	SWI/SNF-non-mutant	SWI/SNF-mutant	*p*
Breast cancer	4.9	9.3	0.001	13.9%	57.1%	0.001	0.0%	0.0%	NA
Cervical cancer	4.7	9.7	0.001	11.7%	40.0%	0.012	1.9%	0.0%	0.999
Colorectal cancer	7.2	44.1	< 0.0001	1.6%	39.6%	< 0.0001	1.2%	33.2%	< 0.0001
Endometrial cancer	6.2	61.6	< 0.0001	14.3%	75.8%	< 0.0001	8.9%	48.5%	< 0.0001
Esophagus cancer	6.5	13.9	< 0.0001	24.1%	75.0%	0.013	0.0%	0.0%	NA
Gallbladder and Biliary tract cancer	6.1	17.6	0.019	16.7%	43.5%	0.063	3.3%	17.4%	0.154
Gastric cancer	4.2	15.3	0.002	7.2%	31.6%	< 0.0001	0.0%	12.3%	0.0004
Kidney cancer	3.5	9.1	0.004	7.4%	28.6%	0.180	0.0%	0.0%	NA
Liver cancer	4.6	12.4	0.003	11.6%	33.3%	0.050	0.0%	6.7%	0.179
Melanoma	3.3	6.8	< 0.0001	3.8%	20.0%	0.042	0.0%	0.0%	NA
Non-small cell lung cancer	5.6	12.7	< 0.0001	18.9%	53.0%	< 0.0001	0.2%	1.1%	0.086
Other	4.0	16.6	< 0.0001	9.1%	51.1%	< 0.0001	1.0%	2.2%	0.462
Ovarian and Fallopian tube cancer	3.7	9.1	< 0.0001	3.6%	18.4%	< 0.0001	1.1%	4.6%	0.059
Pancreatic cancer	4.0	6.7	0.006	3.9%	12.5%	0.203	1.3%	0.0%	0.999
Prostatic cancer	6.9	46.1	0.010	12.5%	33.3%	0.422	6.3%	33.3%	0.298
Small cell lung cancer	9.2	22.1	0.016	58.8%	100.0%	0.521	0.0%	0.0%	NA
Soft tissue sarcoma	2.5	9.4	< 0.0001	4.4%	42.9%	0.0002	2.7%	14.3%	0.093
Urothelial cancer	7.3	15.1	0.005	32.6%	57.9%	0.092	0.0%	5.3%	0.306
Total	5.6	25.8	< 0.0001	44.3%	10.3%	< 0.0001	0.9%	16.0%	< 0.0001

*Mb* megabase, *MSI-H* high microsatellite instability, *SWI/SNF* switch/sucrose nonfermentable, *TMB-H* high tumor mutation burden

### ICI treatment outcomes of patients with SWI/SNF mutations

**Fig. 4:**
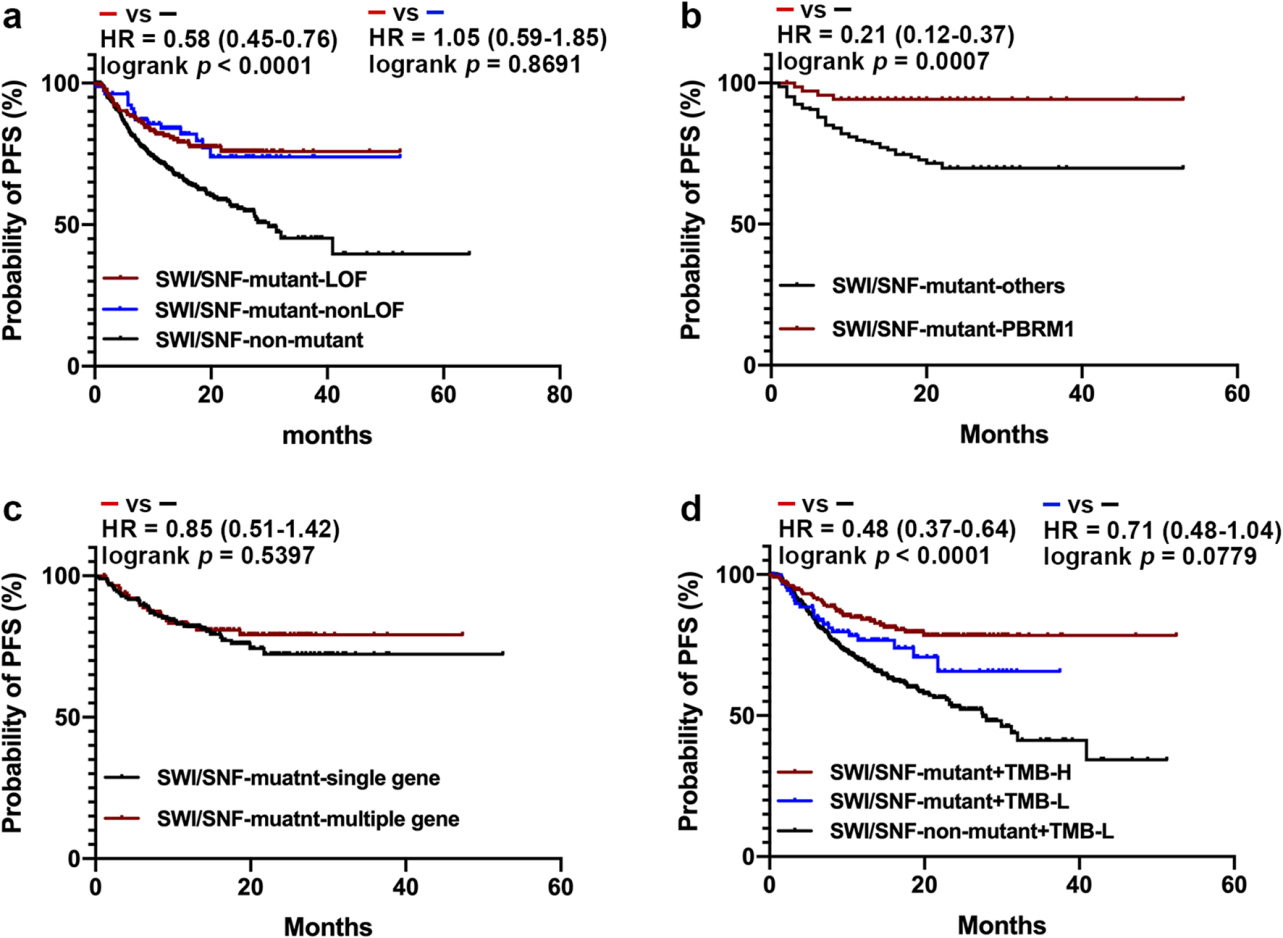
The progression-free survival (PFS) of patients receiving immune checkpoint inhibitor (ICI) treatment in different groups. **a** The PFS of patients receiving ICI treatment in SWI/SNF-mutant-loss-of-function (SWI/SNF-mutant-LOF), SWI/SNF-mutant-non-LOF, and SWI/SNF-non-mutant groups. **b** The PFS of patients treated by ICIs carrying *PBRM1* mutations was significantly longer than that of patients carrying the other SWI/SNF gene mutations. **c** The PFS of patients treated by ICIs carrying mutations in two or more SWI/SNF genes was not significantly different from that of patients with mutations in single gene. **d** The different ICI response of the SWI/SNF-mutant + low tumor mutational burden (TMB-L), the SWI/SNF-non-mutant + TMB-L cohort, and the SWI/SNF-mutant + high tumor mutational burden (TMB-H) cohorts

Over the past few years, pre-clinical and clinical evidence has implicated the SWI/SNF complex as a potential predictor of response to ICIs [6]. For the ICI-treated patients, we observed that the presence of SWI/SNF LOF variants was significantly associated with a longer PFS (not reached [NR] vs. 29.9 months, HR = 0.58 [0.45–0.76]; *p * < 0.0001), and the presence of non-LOF variants was not inferior to the LOF variants (NR vs. NR, HR = 1.05 [0.59–1.87]; *p * = 0.8691; Fig. 4a). The exploration of the predicting significance of each SWI/SNF gene mutation showed that *PBRM1* mutations were associated with a relatively better outcome of ICI treatments than the other SWI/SNF gene mutations (NR vs. NR, HR = 0.21 [0.12–0.37], *p * = 0.0007; Fig. 4b). Specifically, patients carrying mutations at two or more SWI/SNF genes did not show a superior PFS than single gene mutation carriers (NR vs. NR, HR = 0.85 [0.51–1.42], *p * = 0.5397; Fig. 4c). Notably, the prediction value of the SWI/SNF variants increased considerably when the TMB-H status was also considered. In particular, we observed that the SWI/SNF-mutant + TMB-low (TMB-L) cohort showed a numerically but not statistically longer PFS than the SWI/SNF-non-mutant + TMB-L cohort (NR vs. 27.5 months, HR = 0.71 [0.48–1.04], *p* = 0.0779), while that the SWI/SNF-mutant + TMB-H cohort showed a significantly longer PFS than the SWI/SNF-non-mutant + TMB-L cohort (NR vs. 27.5 months, HR = 0.48 [0.37–0.64], *p* < 0.0001; Fig. 4d). Additionally, the survival analysis for individual cancer types suggested that the PFS of the SWI/SNF-mutant group was significantly superior to that of the SWI/SNF-non-mutant group in colorectal cancer (NR vs. NR, HR = 0.33 [0.19–0.59], *p* = 0.0001; Additional file 2: Figure S2a) and gastric cancer (NR vs. 20.6 months, HR = 0.44 [0.19–0.97], *p* = 0.0437; Additional file 2: Figure S2b); the same tendency was significant numerically but not statistically in non-small cell lung cancer (NR vs. 40.9 months, HR = 0.58 [0.33–1.02], *p* = 0.0595; Additional file 2: Figure S2c). The PFS of SWI/SNF-mutant and SWI/SNF-non-mutant groups were not markedly different (Additional file 2: Figure S2d–h) or could not be analyzed owing to the small sample size in the other malignancies.

Regardless of the cancer type, patients in the SWI/SNF-mutant group showed higher ORR (3.32% vs. 0.43%, *p* = 0.0002) and DCR (80.07% vs. 65.57%, *p* < 0.0001) values than their counterparts in the SWI/SNF-non-mutant group. For individual cancer types, SWI/SNF-mutant colorectal cancer (86.27% vs. 67.83%, *p* = 0.0014), gastric cancer (83.33% vs. 55.77%, *p * = 0.0222), and non-small cell lung cancer (85.07% vs. 71.58%, *p * = 0.0324) showed significantly higher DCR values in immunotherapy than their SWI/SNF-non-mutant counterparts (Table 4).

**Table 4:** The overall response rate and disease control rate in the SWI/SNF-mutant and SWI/SNF-non-mutant groups

Cancer type	SWI/SNF-mutant			SWI/SNF-non-mutant			*p* for ORR	*p* for DCR
	n	ORR (%)	DCR (%)	n	ORR (%)	DCR (%)		
Total	301	3.32	80.07	700	0.43	65.57	0.0002	< 0.0001
Breast cancer	4	0	25.00	15	0	40.00	0.9999	0.9999
Cervical cancer	6	0	50.00	25	4.00	64.00	0.9999	0.6526
colorectal cancer	102	4.90	86.27	115	0.87	67.83	0.1016	0.0014
Endometrial cancer	17	0	64.71	16	0	75.00	0.9999	0.7080
Esophagus cancer	6	0	50.00	17	0	47.06	0.9999	0.9999
Gallbladder and Biliary tract cancer	6	0	50.00	11	0	90.91	0.9999	0.0987
Gastric cancer	24	0	83.33	52	0	55.77	0.9999	0.0222
kidney cancer	5	0	100.00	7	0	71.43	0.9999	0.4697
Liver cancer	7	0	100.00	27	0	74.07	0.9999	0.2997
Melanoma	8	12.50	62.50	74	0	50.00	0.0976	0.7130
Non-small cell lung cancer	67	2.99	85.07	190	0.53	71.58	0.1674	0.0324
Other	16	12.50	81.25	62	0	69.35	0.0400	0.5345
Ovarian and Fallopian tube cancer	6	0	66.67	12	0	50.00	0.9999	0.6380
Pancreatic cancer	4	0	100.00	13	0	69.23	0.9999	0.5193
Prostatic cancer	1	0	100.00	5	0	60.00	0.9999	0.9999
Small cell lung cancer	1	0	100.00	12	0	66.67	0.9999	0.9999
Soft tissue sarcoma	4	0	50.00	19	0	52.63	0.9999	0.9999
Urothelial cancer	17	0	76.47	28	0	82.14	0.9999	0.7109

*DCR* disease control rate, *n* number, *ORR* overall response rate, *SWI/SNF* switch/sucrose nonfermentable

### Synthetic lethality involving SWI/SNF members

In recent years, synthetic lethality has attracted considerable attention in oncology, as it may explain the sensitivity of cancer cells to certain inhibitors and provide a new angle for drug development. The previously reported synthetic lethal pairs and effective inhibitors in SWI/SNF-deficient cancers are summarized in Additional file 4: Table S1. These synthetic lethal interactions can be classified under four main categories. (a) Two subunits within the SWI/SNF complex. For example, the BRD2 inhibitor, JQ1, can suppress *ARID1A*-deficient ovarian clear cell cancer cells because BRD2 inhibition decreases *ARID1B* transcription [48]. (b) One SWI/SNF subunit with its competitor. Contrary to the chromatin relaxation-inducing function of the SWI/SNF complex, polycomb repressive complex 2 (PRC2), whose enzymatic catalytic subunit is the methyltransferase, EZH2, promotes chromatin compaction via histone H3 K27 trimethylation (H3K27me3). Thus, the inhibition of EZH2 using tazemetostat or GSK126 causes synthetic lethality in *ARID1A*-, *SMARCA4*-, *SMARCB1*-, *PBRM1*-deficient cancers [49–54]. (c) Targeting the functions of the SWI/SNF complex. The SWI/SNF chromatin remodeling complex functions in DNA double-strand break repair, transcription, replication, chromosomal segregation, and in several metabolic pathways. Therefore, SWI/SNF-deficient cancers are vulnerable to the inhibition of homologous recombination repair factor, PARP1 [20, 49], cell cycle regulator, cyclin-dependent kinase (CDK)4/CDK6 [28, 56], DNA replication checkpoint factor, ATR [57], chromosomal segregation factor, Aurora kinase A [58], and oxidative phosphorylation [59] and glutathione [60] pathways. (d) Others: PD-1/PD-L1 inhibitors have synthetic lethal effects in *ARID1A*- and *PBRM1*-deficient cancers [24, 61].

## Discussion

Throughout development, chromatin architecture undergoes dynamic changes that are critical for enhancer activation and gene expression. The mammalian SWI/SNF chromatin remodeling complex plays a crucial role in cellular and tissue development, and SWI/SNF subunits have been implicated as suppressors in a variety of human cancers [7, 62]. In the present study, NGS data corresponding to 4591 solid tumors, covering 18 types of malignancies, were retrospectively integrated to depict the spectrum of SWI/SNF variations. The SWI/SNF genes, *ARID1A*, *ARID1B*, *ARID2*, *SMARCA4*, *SMARCB1*, and *PBRM1* were mutated in up to 21.8% of all the cancers, and SWI/SNF mutation carriers had significantly higher TMB values as well as higher TMB-H and MSI-H proportions than their SWI/SNF-non-mutant counterparts in several malignancies. *ARID1A* was the most frequently altered SWI/SNF gene and *ARID1A* D1850fs was identified as a relatively hot spot. Clinically, SWI/SNF mutations were found to be closely associated with a better response to ICI treatments in colorectal cancer, gastric cancer, and non-small cell lung cancer. The PFS was not significantly different in SWI/SNF-mutant and -non-mutant groups in other cancers, which might be due to the relatively small number of cases involved in our study. Given that most SWI/SNF mutations were dispersed along the full length of each gene, NGS showed potential as the most suitable strategy for detecting SWI/SNF alterations.

ARID1A/B (BAF250a/b) contains two primary domains: an N-terminal AT-rich interacting domain (ARID, residues 1017–1104) and a C-terminal domain DUF3518, also annotated as BAF250_C (residues 1975–2231). Specifically, ARID, which is a conserved helix-turn-helix motif-containing domain, plays a role in recruiting SWI/SNF to the target gene promoters, whereas the function of the BAF250_C domain, which contains motifs, such as NES and LXXLL-motif, that putatively mediate protein–protein interactions, is still unknown [63]. A TCGA database search revealed that the R1989* nonsense mutation in the DUF3518 domain is a hotspot mutation of *ARID1A* across cancers [64]. In this study, we observed that R1989* was captured less frequently than D1850Tfs*33 and D1850Gfs*4 (Fig. 2a), possibly because the study included a very high proportion of colorectal cancer cases, and reportedly, D1850fs is an *ARID1A* hot spot in colorectal cancer [65]. Additionally, the DUF3518 domain of *ARID1A* was found to be functionally necessary to antagonize EZH2, and both the R1989* variant and the deletion of the DUF3518 domain could not rescue EZH2-mediated IFN-γ signaling gene repression in ARID1A-knockout ovarian cancer cells [66]. D1850Tfs*33 and D1850Gfs*4, which are frameshift truncating mutations, brought about the loss of more amino acids than did R1989*. Therefore, we concluded that D1850Tfs*33 and D1850Gfs*4 might exert their functions via the deletion of the DUF3518/BAF250_C domain. The previously reported V1067G mutation, which destabilizes the ARID domain, was not detected in any of the cases included in this study [67].

Somatic mutations in *SMARCA4* and/or BRG1 (Brahma-related gene 1) loss are present in a subset of non-small cell lung carcinomas with distinct morphological features, harboring less *EGFR* mutations, but more *KRAS*, *STK11*, and *KEAP1* mutations [68, 69]. In a study on lung cancer, the genes most frequently co-mutated with *SMARCA4* were *TP53* (56%), *KEAP1* (41%), *STK11* (39%), *KRAS* (36%), and *EGFR* (14%) [68]. Among the 58 cases of lung cancer with *SMARCA4* LOF mutations in our study, the mutation rates corresponding to the above hot genes were almost consistent with the previously reported rates of 74.1%, 31.0%, 24.1%, 20.7%, and 15.5% for *TP53*, *KEAP1*, *STK11*, *KRAS*, and *EGFR*, respectively. In this subset, 10 of 11 patients treated with ICIs attained a stable disease state, with only one patient showing disease progression (median PFS = 17.6 month). Thus, the detection of a *SMARCA4* variant via NGS was useful not only in defining the particular pathological diagnosis but also in providing important clues for the choice of treatment for SMARCA4-deficient lung cancer.

LOF variants of the SWI/SNF complex can influence the response to ICIs by increasing the infiltration of CD8 + T cells, enhancing the cytotoxicity of T cells [70], or by creating an immune-responsive milieu [24]. In the current study, the PFS of patients with SWI/SNF LOF mutations was not significantly longer than that of the SWI/SNF non-LOF mutation carriers, suggesting that at least a proportion of the SWI/SNF non-LOF mutations, the most of which are missense mutations, occurring at pivotal sites might be functional. However, further studies are required to provide additional evidence for more accurate interpretation using bioinformatics. The patients carrying mutations of two or more SWI/SNF genes did not show better responses to the ICI therapy than those with single gene mutations, indicating that the increase in the number of SWI/SNF complex mutated genes may not directly cause an accumulative effect. The immunotherapeutic effect-predicting biomarker section of several commercially available NGS panels includes positively related gene variations, such as TMB-H [71], MSI-H [72], inactivating mutations of mismatch repair-related genes (*MLH1*, *MSH2*, *MSH6*, *PMS2*) [73], homologous recombination repair-related genes (*ATM*, *ATR*, *BRCA1/2*, *CHEK1*, *FANC*A, *PALB2*, etc.) [74], and *POLE* and *POLD1* mutations [75] as well as negatively related gene variations, including inactivating mutations of *PTEN* [76], *B2M* [77], *JAK1/2* [78], *DNMT3A* [79], *STK11* [80], copy number gain of *MDM2/4* [79], and *CCND1* [81]. Given that patients with SWI/SNF variations showed significantly longer PFS than their SWI/SNF-non-mutant counterparts (HR, 0.56 [95% CI 0.44–0.72]; *p * < 0.0001), the SWI/SNF variations could be added to the list of positively predicting biomarkers for immunotherapeutic effects.

Abou Alaiwi et al. [6] also investigated the relationship between SWI/SNF complex gene variations and the ICI response by analyzing data from seven types of solid tumors, whereas we included a large patient cohort from China involving more than 18 cancer types. The previous study also excluded missense mutations from their study, whereas we stratified missense mutations into LOF and non-LOF mutations using two outstanding in silico predicted ensemble scores, MetaLR and MetaSVM, and showed that non-LOF mutations were not inferior to the LOF mutations in predicting PFS. Additionally, the previous study found that only patients with renal cell carcinoma and SWI/SNF-LOF mutations showed significantly improved survival in the cohort from Dana Farber Cancer Institute, which was mostly driven by *PRBM1*. Similarly, we found that *PBRM1* mutations were associated with a better outcome of ICI treatments than the other SWI/SNF gene mutations (Fig. 4b). We also agreed with Abou Alaiwi et al. that loss of the SWI/SNF complex cannot be used as a pan-cancer biomarker of clinical benefits from ICIs. The current study demonstrated SWI/SNF complex variations were tightly associated with superior ICI response in several solid tumors, such as colorectal cancer, gastric cancer, and non-small cell lung cancer, especially when combined with TMB-H status. This may be caused by the involvement of a large number of colorectal cancer and non-small cell lung cancer cases as well as the missense mutations classification strategy in our study, and two different cohorts, respectively, from Dana Farber Cancer Institute and Memorial Sloan Kettering Cancer Center using two different NGS detection pipelines in their study.

The high mutation rate of the SWI/SNF complex across all cancers highlights its potential as a target for tissue-agnostic drugs. Synthetic lethality occurs when a combination of deficiencies in two genes leads to cell death, whereas deficiency in only one gene results in a viable phenotype [50]. Notably, PARP inhibitors targeting *BRCA1/2*-mutant tumors represent a notable example of such synthetic lethality [82]. A series of inhibitors, ranging from chemical probes to FDA-approved drugs, that target the synthetic lethal partners of SWI/SNF members have been shown to exhibit clear therapeutic effects in several cancers [20, 21, 25, 48–60, 81–105]. Furthermore, an overview of the possible biological functions and downstream signaling pathways using the GO database suggested that SWI/SNF genes and covariant genes were enriched in the PI3K signaling pathway (Additional file 3: Figure S3). Reportedly, *ARID1A*-deficient gastric cancer cells are vulnerable to the AKT inhibitor, GSK690693, and the addition of GSK690693 possibly potentiates the suppressive function of conventional chemotherapy [105]. Accordingly, the therapeutic effect of AKT inhibitors in cancers with SWI/SNF deficiencies is promising and should be explored further.

By integrating NGS data from a large real-world patient cohort, this study offers a detailed overview of the genomic alterations in SWI/SNF complex genes in various cancer types, and reveals the significant associations between SWI/SNF variants and TMB, MSI, and response to ICI treatment in colorectal cancer, gastric cancer, and non-small cell lung cancer; this could be of great significance in molecular screening and translational research. We mainly focused on six SWI/SNF genes that mutate with high frequencies, other SWI/SNF subunits, such as SMARCC1, SMARCC2, SMARCD1/D2/D3, SMARCE1, and ACTL6A/B, which are reported to be mutated infrequently in primary tumors [7], were not investigated since the targeted sequencing panels did not contain all the SWI/SNF complex members; we could not, therefore, assess the association of the other SWI/SNF complex members with the ICI response. The molecular functions and relevant signaling mechanisms involving the SWI/SNF variations were not investigated experimentally, and warrant further exploration.

## Conclusions

SWI/SNF complex genes are frequently mutated in a wide range of cancers and are closely associated with TMB-H, MSI-H, and superior responses to ICIs in colorectal cancer, gastric cancer, and non-small cell lung cancer. Therefore, the detection and interpretation of genomic alterations in the SWI/SNF complex using NGS could provide new predictors of immunotherapeutic effects as well as useful data for translational research.

## Supplementary Information

**Additional file 1: Fig. S1** The distributions of variant allele frequencies (VAFs) of *ARID1A*, *ARID1B*, *ARID*2, *PBRM1*, *SMARCA4*, and *SMARCB1*. The median VAFs of the above genes were 16.1%, 13.4%, 13.3%, 17.2%, 15.2%, and 16.7%, respectively. Red solid line, median; black dotted line, quartiles.

**Additional file 2: Fig. S2** The progression-free survival (PFS) of patients receiving immune checkpoint inhibitor (ICI) treatment based on cancer types. The survival analysis was performed for individual cancer types that contained at least 10 cases in the SWI/SNF-mutant or SWI/SNF-non-mutant groups. The PFS of the SWI/SNF-mutant group was significantly superior to that of the SWI/SNF-non-mutant group in colorectal cancer (a) and gastric cancer (b), the same tendency was significant numerically by not statistically in non-small cell lung cancer (c). The PFS of SWI/SNF-mutant and SWI/SNF-non-mutant were not markedly different in melanoma (d), soft tissue sarcoma (e), urothelial cancer (f), endometrial cancer (g) and other cancers (h).

**Additional file 3: Fig. S3** The signaling pathway enrichment of the variated genes in the SWI/SNF-mutant tumors by GO analysis. The GO analysis was performed on all the mutated genes in 1001 SWI/SNF-mutant samples.

**Additional file 4: Table S1.** Synthetic lethal interactive pairs and chemical inhibitors involving SWI/SNF members.

## Data Availability

The datasets supporting the conclusions of this article are available in the Research Data Deposit repository (No. RDDA2021338857, http://www.researchdata.org.cn/), and are available from the corresponding author on reasonable request.

## Abbreviations

DCR: Disease control rate
ICI: Immune checkpoint inhibitor
LOF: Loss-of-function
MSI-H: High microsatellite instability
Mb: Megabase
NGS: Next-generation sequencing
ORR: Overall response rate
PFS: Progression-free survival
SWI/SNF: SWItch/sucrose nonfermentable
TMB-H: High tumor mutational burden
TMB-L: Low tumor mutational burden
