# Supplementary figures and images for: SWI/SNF complex gene variations are associated with a higher tumor mutational burden and a better response to immune checkpoint inhibitor treatment: a pan-cancer analysis of next-generation sequencing data corresponding to 4591 cases
Source: Cancer Cell Int. 2022 Nov 12;22:347. doi: 10.1186/s12935-022-02757-x (PMC9652899; doi:10.1186/s12935-022-02757-x)

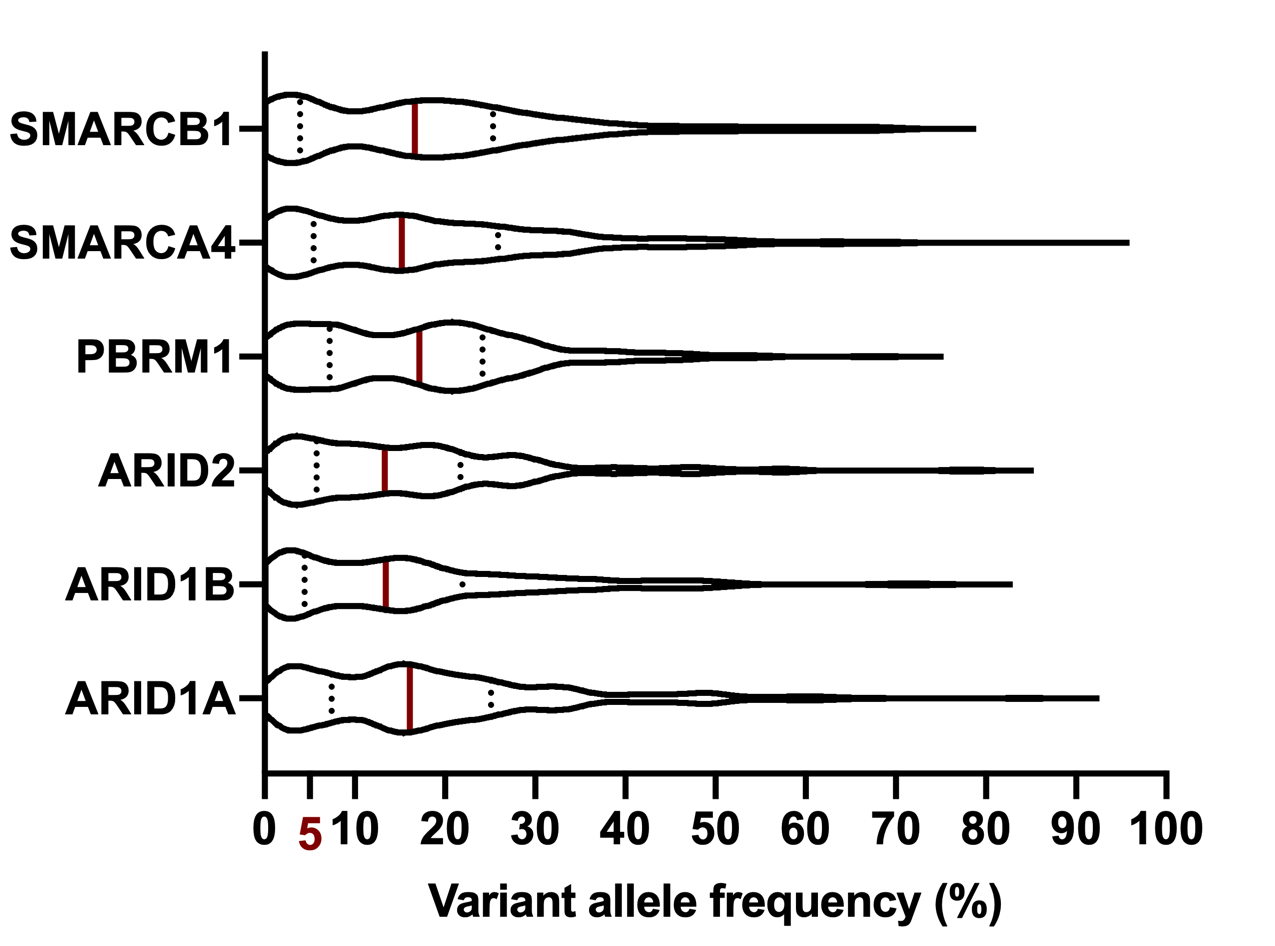

Supplement: Supplementary file 1 — Additional file 1: Fig. S1 The distributions of variant allele frequencies (VAFs) of ARID1A, ARID1B, ARID2, PBRM1, SMARCA4, and SMARCB1. The median VAFs of the above genes were 16.1%, 13.4%, 13.3%, 17.2%, 15.2%, and 16.7%, respectively. Red solid line, median; black dotted line, quartiles. [file 12935_2022_2757_MOESM1_ESM.tiff]

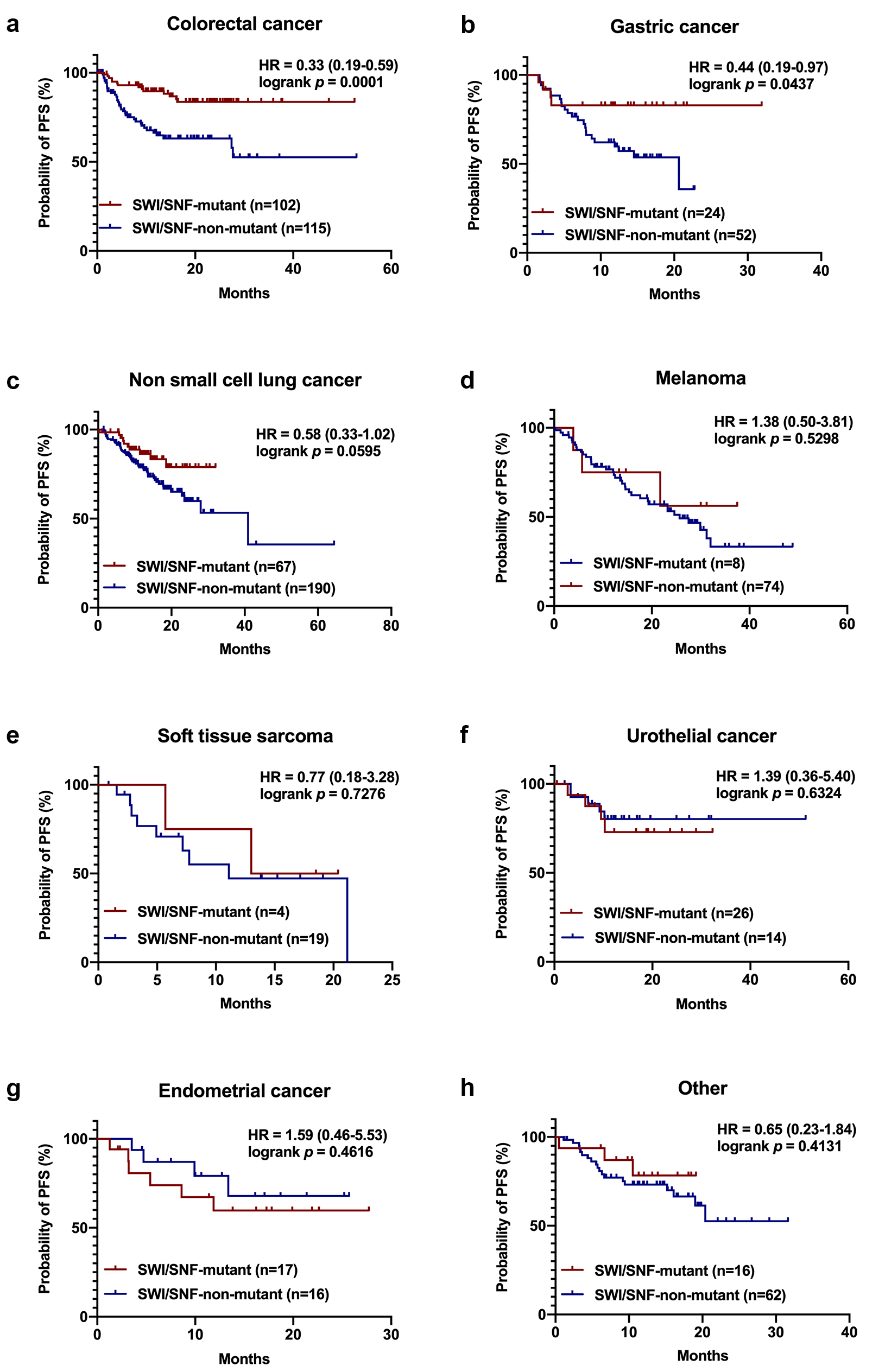

Supplement: Supplementary file 2 — Additional file 2: Fig. S2 The progression-free survival (PFS) of patients receiving immune checkpoint inhibitor (ICI) treatment based on cancer types. The survival analysis was performed for individual cancer types that contained at least 10 cases in the SWI/SNF-mutant or SWI/SNF-non-mutant groups. The PFS of the SWI/SNF-mutant group was significantly superior to that of the SWI/SNF-non-mutant group in colorectal cancer (a) and gastric cancer (b), the same tendency was significant numerically by not statistically in non-small cell lung cancer (c). The PFS of SWI/SNF-mutant and SWI/SNF-non-mutant were not markedly different in melanoma (d), soft tissue sarcoma (e), urothelial cancer (f), endometrial cancer (g) and other cancers (h). [file 12935_2022_2757_MOESM2_ESM.tif]

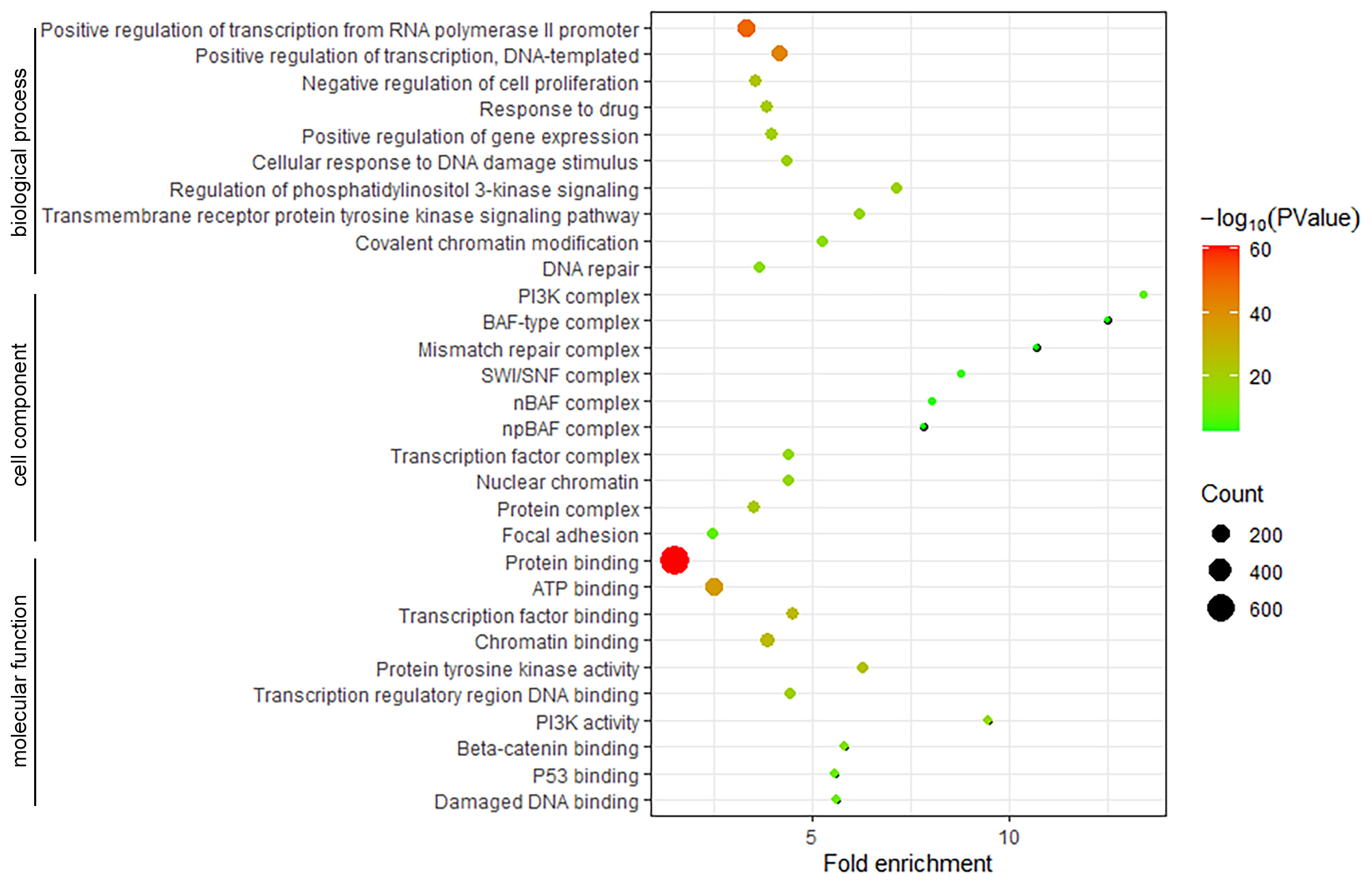

Supplement: Supplementary file 3 — Additional file 3: Fig. S3 The signaling pathway enrichment of the variated genes in the SWI/SNF-mutant tumors by GO analysis. The GO analysis was performed on all the mutated genes in 1001 SWI/SNF-mutant samples. [file 12935_2022_2757_MOESM3_ESM.tif]
