# Supplementary material for: SWI/SNF complex gene variations are associated with a higher tumor mutational burden and a better response to immune checkpoint inhibitor treatment: a pan-cancer analysis of next-generation sequencing data corresponding to 4591 cases
Source: Cancer Cell Int. 2022 Nov 12;22:347. doi: 10.1186/s12935-022-02757-x (PMC9652899; doi:10.1186/s12935-022-02757-x)
Supplement: Supplementary file 4 — Additional file 4: Table S1. Synthetic lethal interactive pairs and chemical inhibitors involving SWI/SNF members. [file 12935_2022_2757_MOESM4_ESM.docx]

**Table S1** Synthetic lethal interactive pairs and chemical inhibitors involving SWI/SNF members.

| SWI/SNF member | synthetic lethal partner | Inhibitors | Disease | Reference |
| --- | --- | --- | --- | --- |
| ARID1A | ATR | VE-821, VX-970 (M6620), AZD6783 | Breast cancer, ovarian cancer, colorectal cancer | 57 |
|  | AURKA | TCS-7010 | Colorectal cancer | 58 |
|  | BIRC5/Survivin | YM-155 | Gastric cancer | 92 |
|  | BRD2 | JQ1/ iBET762 | Ovarian cancer | 48 |
|  | CARM1, p53 | TP064+Nutlin-3 | Gastric cancer | 93 |
|  | CCNE1 | NA | Ovarian cancer | 88 |
|  | EZH2 | GSK126, GSK343, EPZ005687 | Ovarian cancer, gastric cancer | 49, 50 |
|  | GSH | buthionine sulfoximine, APR-246 | Ovarian cancer, gastric cancer | 60 |
|  | HDAC6 | ACY1215 (rocilinostat), SAHA | Ovarian cancer | 94, 95 |
|  | mTOR | RAD001 | Gastric cancer | 96 |
|  | PARP1 | olaparib, rucaparib, and veliparib | Breast cancer, colorectal cancer | 55 |
|  | PD-1/PD-L1 | Nivolumab | Endometrial cancer | 97 |
|  | PI3K/AKT | GSK690693, BKM120 | Gastric cancer | 98, 105 |
|  | YES1/SRC | Dasatinib (BMS-354825) | Ovarian cancer | 86 |
| SMARCA4 | AURKA | VX-680 | Lung cancer | 99 |
|  | CDK4/CDK6 | Palbociclib, Abemaciclib, Ribociclib | Lung cancer, ovarian cancer | 28, 56 |
|  | EZH2 | GSK126, Tazemetostat, CPI-169 | Ovarian cancer | 51, 83–85 |
|  | KDM6A | GSK-J4 | Lung cancer, ovarian cancer | 90 |
|  | OXPHOS | IACS-010759 | Lung cancer | 59 |
|  | PTEN | PFI-3 | Prostate cancer | 100 |
| PBRM1 | EZH2 | L501-1669 | Renal cell cancer | 54 |
|  | PD-1/PD-L1 | Nivolumab | Renal cell cancer | 24 |
| ARID2 | PARP1 | veliparib | Lung cancer | 23 |
| SMARCB1 | AURKA | Alisertib (MLN8273) | Rhabdoid tumors | 101 |
|  | CCND1 | Flavopiridol | Rhabdoid tumors | 102 |
|  | EZH2 | Tazemetostat | Pooly differentiated chordoma | 52, 53 |
|  | GLI1 | arsenic trioxide | Rhabdoid tumors | 103 |
|  | MDM2/MDM4 | Idasanutlin, ATSP-7041 | Rhabdoid tumors | 91 |
|  | UBE2C | Bortezomib/MLN2238 | Renal medullary carcinoma | 104 |

OXPHOS: oxidative phosphorylation; PD-1: programmed death-1; PD-L1: programmed death-ligand 1; SWI/SNF: SWItch/sucrose nonfermentable.
